# Fuzzy-Based Hybrid Control Algorithm for the Stabilization of a Tri-Rotor UAV

**DOI:** 10.3390/s16050652

**Published:** 2016-05-09

**Authors:** Zain Anwar Ali, Daobo Wang, Muhammad Aamir

**Affiliations:** 1 College of Automation Engineering, Nanjing University of Aeronautics and Astronautics, Nanjing 210016, China; dbwangpe@nuaa.edu.cn; 2 Electronic Engineering Department, Sir Syed University of Engineering and Technology, Karachi 75300, Pakistan; muaamir5@yahoo.com

**Keywords:** Unmanned Aerial Vehicle, Tri-Rotor UAV, RST controller, fuzzy hybrid controller

## Abstract

In this paper, a new and novel mathematical fuzzy hybrid scheme is proposed for the stabilization of a tri-rotor unmanned aerial vehicle (UAV). The fuzzy hybrid scheme consists of a fuzzy logic controller, regulation pole-placement tracking (RST) controller with model reference adaptive control (MRAC), in which adaptive gains of the RST controller are being fine-tuned by a fuzzy logic controller. Brushless direct current (BLDC) motors are installed in the triangular frame of the tri-rotor UAV, which helps maintain control on its motion and different altitude and attitude changes, similar to rotorcrafts. MRAC-based MIT rule is proposed for system stability. Moreover, the proposed hybrid controller with nonlinear flight dynamics is shown in the presence of translational and rotational velocity components. The performance of the proposed algorithm is demonstrated via MATLAB simulations, in which the proposed fuzzy hybrid controller is compared with the existing adaptive RST controller. It shows that our proposed algorithm has better transient performance with zero steady-state error, and fast convergence towards stability.

## 1. Introduction

One of the best inventions of today’s era is the small flying machine commonly called a UAV. This research is dedicated to such types of UAVs, which are commonly used in the monitoring of disaster management and military operations, as well as small indoor activities [1,2,3]. The research on UAVs is based on the different knowledge banks of aeronautics, signal processing, and control automation. For this research, multiple hardware-based tests are performed to design the best flying machines with precise control mechanisms.

The current trend is focused on the design of advanced, lightweight, and perfect UAVs that can be operated in any disastrous situations over remote areas. UAVs are classified as either fixed-wing or rotary wing [4]. Rotor-based UAVs are multiple input and multiple output (MIMO) multivariable systems [5]. Rotorcraft have a great advantage over fixed-wing aircraft with respect to various applications, like vertical takeoff and vertical landing (VTOL) capability and payloads. Rotor-based UAVs include many types, such as bi-rotor, tri-rotor, quad-rotor and hex-rotor [6]. Moreover, a tri-rotor UAV with VTOL ability is considered in this paper.

Real-world application of UAVs require intense hardware testing. Before the experimental testing of our proposed algorithm in the real world, we have to simulate the numerical nonlinear simulations for the Euler angles, control commands, rotational velocities, and translational velocities [7]. In this research, our main concern is to rectify the error which occurs in a yaw moment due to the unpaired reaction of the rotors, thereby producing torque. Brushless direct current (BLDC) motors are installed in the triangular frame of the tri-rotor craft to nullify the tilt angle moment.

The dynamics of the UAV are highly nonlinear and multi-variable, with a lot of parameter uncertainties, many effects to which a potential controller has to be robust. The aerodynamics of the actuator blades (flapping of blade and propeller), inertial torques (angular speed of propellers), and gyroscopic effects (which change the orientation of the UAV) are found in [8]. The redundancy in the rotors of a UAV formulates them towards a set of partial collapses. Although the maneuverability and performance will probably be condensed in the case of such a collapse, it is required that a controller stabilizes the system and tolerates reduced mode functions, such as safe arrival, steady hover, *etc*. [9,10].

Previously, many control methods were used for the stabilization of UAVs, including the conventional proportional integral derivative (PID) controller, fuzzy controller, adaptive controller, and so on [11]. For controlling the parameters of a UAV an adaptive controller has a capability to give good performance in the presence of model and parametric uncertainties, while MRAC is concerned with the vibrant reaction of the controlled system to asymptotic convergence. It follows the reference system in spite of parametric model uncertainties in the system [12].

In [13] the proposed MRAC for controlling the dynamics of a quad-rotor in the presence of actuator uncertainties was considered to enhance an existing linear controller, offering autonomous waypoint following. The stability of the adaptive controller was ensured by the Lyapnauv theorem and, in a nonlinear structure, the algorithm is applied for indoor flight test.

In [14] the hybrid control scheme to fault tolerant control (FTC) for a quad-rotor aircraft in the presence of faults in their rotors during the flight have been explored and tested on the MRAC algorithm and a gain-scheduled PID (GS-PID) control. MRAC and GS-PID are used in collaboration with a linear quadratic regulator (LQR) to control the attitude of the UAV. MRAC is based on MIT rules for controlling the height and other parameters of a Qball-X4 Quad-Rotor aircraft.

Takagi-Sugeno fuzzy rules were previously used in [15,16] to control the nonlinear behavior of the vehicle. On the other hand, in [17], a twin controller approach that consists of a backstepping controller to control the nonlinear dynamics of the system and linguistic logic rules of a fuzzy logic controller (Mamdani) is used to control the attitude of tilt of a tri-rotor UAV. In [18] a dual controller approach with an adaptive fuzzy sliding mode controller is used to control the mini UAV, in which sliding mode control is utilized to control the nonlinear behavior of the UAV, and then fuzzy logic rules are implemented on it. The hybrid controller approach was also addressed in [19] in which a fuzzy-PID controller with a PSO algorithm is applied on tri-rotor dynamics.

Hwoever, in this paper, we proposed a fuzzy hybrid controller consisting of a RST with MRAC, based on MIT rules working as a main controller in the model to deal with the nonlinear system. We compare the performance of our proposed fuzzy hybrid controller with the robust adaptive RST controller of [20].

Moreover, the adaptive gains of the RST controller are (*i.e.*, regulation gain $“G_{R}”$, pole-placement gain $G_{S},$ and tracking gain $“G_{T}”$) tuned by a fuzzy logic controller (Mamdani technique). This means that our main controller is a RST with MRAC based on MIT rules, and for the tuning purpose we use the Mamdani fuzzy logic controller. We have to implement the gains of RST by adding fuzzy logic between uniform scales of membership functions. It shows the best results as compared to the adaptive RST controller [21,22].

In this paper, we are incorporating RST controller with our proposed system in two separate ways. First, the system is undergoes through Robust adaptive RST controller, after that we use Fuzzy-Hybrid based MIT algorithm and then conclude the results by taking the difference of robustness. 

The core contributions in this research are as follows: (1) a novel fuzzy-based adaptive robust RST controller is derived by accumulating the MIT rule in the control law to remove the model disturbance and to derive the steady-state error to zero; (2) the proposed controller uses the angular responses as an input control command, which shows more accurate and practical insight in the real world; (3) in spite of the model disturbance, the close loop system error converges to zero, proved in Theorem 2; and (4) lastly, the polynomial characteristic solution is based on the Diophantine equation while least square estimation is used to check system stability and proved in Theorem 3.

The breakup of this paper is structured as follows. The system modeling, dynamic representation of a tri-rotor UAV, and main engine model is discussed in Section 2. Section 3 demonstrates the dynamic control strategies and the control algorithm of the UAV. Moreover, the simulation results and discussions are discussed in Section 4. Lastly, Section 5 states the conclusions. 

## 2. System Model and Preliminaries

### 2.1. Tri-Rotor Modeling

The equation of motion of a rigid body is defined by Newton’s second law of motion [23,24]. Linear and angular forces change with respect to the timeframe, called the initial reference frame, in which the UAV has a similar velocity, force components, and moments, which are used to develop the six degrees of freedom nonlinear equations of motion. The nonlinear aerodynamic forces, aerodynamic moments, rotation motion, and translational motion of a UAV are defined by using differential Equations (1)–(4).

Equations of aerodynamic force:

(1) $$\begin{array}{c} F_{X}-\text{mg sin}\theta =m\left(\dot{u}+\text{qw}-\text{rv}\right)\\ F_{Y}+\text{mg cos}\theta \text{ sin}\varphi =m\left(\dot{v}+\text{ru}-\text{pw}\right)\\ F_{Z}+\text{mg cos}\theta \text{ cos}\varphi =\text{ m}\left(\dot{w}+\text{pv}-\text{qu}\right) \end{array}$$

Equations of aerodynamic moments:

(2) $$\begin{array}{c} L=I_{x}\dot{p}-{\text{ I}}_{\text{xz}}\dot{\text{r }}+\text{qr }\left(I_{z}-{\text{ I}}_{y}\right)-{\text{ I}}_{\text{xz}}\text{pq}\\ M=I_{y}\dot{q}+\text{rp }\left(I_{x}-{\text{ I}}_{z}\right)+{\text{ I}}_{\text{xz}}\left(p^{2}-{\text{ r}}^{2}\right)\\ N=-I_{\text{xz}}\dot{p}+I_{z}\dot{\text{r }}+\text{pq }\left(I_{y}-{\text{ I}}_{x}\right)+{\text{ I}}_{\text{xz}}\text{qr} \end{array}$$

Rotational rates:

(3) $$\begin{array}{c} p=\dot{\varphi }-\dot{\psi }\text{ sin}\theta \\ q=\dot{\theta \;}\text{cos}\varphi +\dot{\psi }\text{ cos}\theta \text{ sin}\varphi \\ \text{r }=\dot{\psi }\text{ cos}\theta \text{ cos}\varphi -\dot{\theta }\text{ sin}\varphi \end{array}$$

Euler angles and body angular velocities:

(4) $$\begin{array}{c} \dot{\theta }=\text{ q cos}\varphi -\text{r sin}\theta \\ \dot{\varphi }=\text{ p}+\text{q sin}\varphi \text{ tan}\theta +\text{ r}\cos\varphi \text{ tan}\theta \\ \dot{\psi }=\;(\text{q sin}\varphi +\text{r cos}\varphi )\text{sec}\theta \end{array}$$

The four control commands of the tri-rotor UAV are (Col, Lat, Lon, Ped), which is similar to the conventional helicopter, in which Col is Collective, Lat is Lateral, Lon is Longitudinal, and Ped is pedal. (Col, Lat) are used to control the roll rate, (Lon) control the pitch rate and (Ped) controls the yaw rate of a UAV and tilt angle by using the parameter “∝” [25,26]. (p, q, r) and (U, V, W) are the rotational velocity and translational velocity of the coordinate system. (L, M, N) and (φ, θ, ψ) are the external moments and rotational angles of a fixed body frame.

**Figure 1:**
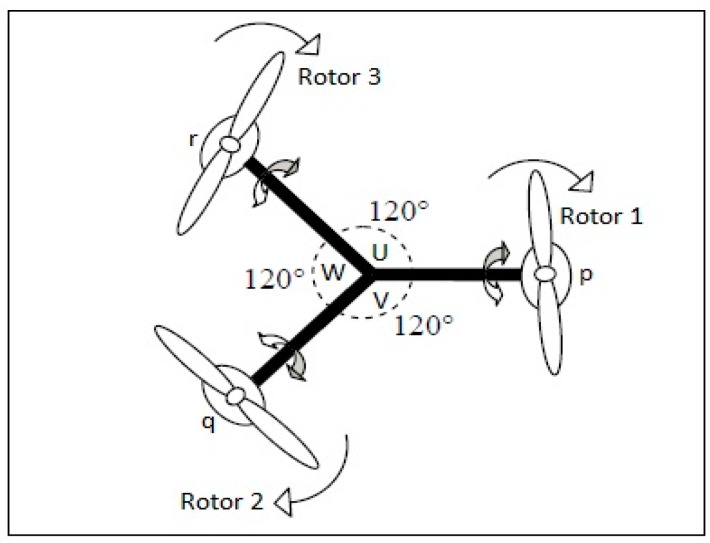
Top view of a tri-rotor UAV along with rotational and transitional rates.

**Figure 2:**
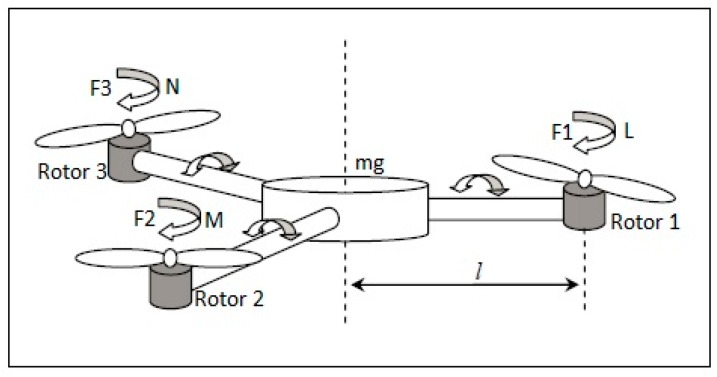
3-D view of a tri-rotor UAV along with aerodynamic moment components.

A tri-rotor aerial vehicle exhibits many physical effects, like inertial torque, effects of aerodynamics, effects of gravity, effects of gyroscope and frictional effects, *etc*. In the presence of these physical effects it is quite difficult to design a controller which can easily handle all of the physical effects and stabilize the UAV in a fair amount of time, because it has six degree of freedom (6-DOF) with a highly-nonlinear, multivariable, under-actuated, strongly-coupled model with the rotors, as shown in Figure 1 and Figure 2 taken from the design of Mohamed MK [27]. 

The tri-rotor UAV, including translational and rotational subsystems, needs a vibrant strategy of nonlinear sequential control in the 6-DOF model. This research addresses error controlling in a tri-rotor aircraft by managing the torque produced by unpaired rotor reactions. To overcome this issue, implementation of different designs has been made with the help of BLDC motors, which actually control the nullifying angle. This method is useful for quicker turn by tilting the rotor’s axis.

### 2.2. Dynamic Representation of a Tri-Rotor UAV

The orientation of the UAV is explained by Euler angles having altitude, roll (θ), pitch (φ), and yaw (Ψ) control and it rotates along (x, y, z) axes, respectively. The translational and rotational movement of the tri-rotor UAV into the dimensional space and dynamics of the rigid body derive from Newton’s law. Moreover, moments, forces, velocity components, and aerodynamic components are described in Table 1.

The overall system configuration is defined in Figure 2, where “L” is the distance from the center of the body frame to all three rotors, labelled as L1, L2, and L3. The rotor forces are f1, f2, f3, and the rotor torque is defined as $\tau_{1},\;\tau_{2}$, and $\tau_{3},$ respectively. The angular velocity of the system is “ρ”.

### 2.3. Main Engine (Electric Motors)

Brushless Direct Current (BLDC) motors can be used as a main engine of the UAV to achieve required electric propulsion in the system. BLDC motors found their application in the field of robotics, space crafts and medical devices, due to higher torque speed features, greater performance, minimum repairs and variable degree of speed [28]. Generally BLDC is more costly than simple DC motors, due to its better efficiency and reliability [29]. The electrical and mechanical equations of BLDC are given below.

(5) $$V=\text{Ri}+\left(L-M\right)\frac{\text{di}}{\text{dt}}+E$$

(6) $$E=K_{e}\omega_{m}\text{ F}\left(\theta_{e}\right)$$

(7) $$T=K_{t}i_{a}F\left(\theta_{e}\right)$$

(8) $${\text{ T}}_{e}=\text{J }\frac{d^{2}\theta_{m}}{{\text{dt}}^{2}}+\beta \frac{{\text{d}\theta }_{m}}{\text{dt}}$$

(9) $$\theta_{e}=\frac{P}{2}\theta_{m}$$

(10) $$\omega_{m}=\;\frac{{\text{d}\theta }_{m}}{\text{dt}}$$

In which, V is the applied voltage; R is the total resistance; E is the back electromagnetic force; L is the total inductance of the motor; M is the mutual inductance of the motor; $\omega_{m}$ is the angular speed of the motor; $\theta_{e}$ is the rotation angle of the motor; $T_{e}$ is the electrical torque produced by the motor; and $K_{e}$ is the back-EMF constant.

## 3. Designing of Controller

### 3.1. Tri-Rotor Dynamic Control Strategies

The flight dynamics and control strategy of a tri-rotor UAV is the same as traditional aircraft. The placement or orientation of flight dynamics control is a product of roll, pitch, and yaw. The control scheme of the UAV includes altitude, roll, pitch, yaw, and tilt angle control, having a major role for the displacement control the parameters of the system.

*Altitude Control Mechanism:* To achieve the desired altitude, the speed of all rotors must be same ρ1 = ρ2 = ρ3. Increasing the speed of all rotors constantly will eventually raise the altitude of the UAV, such that the angular velocity of motors becomes equal.

*Roll Control Mechanism:* Roll control is achieved by regulating the front rotors speed. Decreasing rotor 1 velocity, rolls the system to the left and rotor 2 rolls the system towards right-side. Roll control has two conditions.

- When moving clockwise roll ρ2 >ρ1 >ρ3.
- When moving counter-clockwise roll ρ2 <ρ1 <ρ3.

*Pitch Control Mechanism:* Regulating the speed of Rotor 1 and rearward rotors will change the pitch. The system pitches downward if the speed of Rotors 1 and 2 decreases while Rotor 3 speed keeps rising. If we decrease the speed of Rotor 3 and increase the speed of Rotors 1 and 2, the UAV pitch rises and fly flight reverses. Pitch control also has two conditions:

- When nose-up ρ2 =ρ3 >ρ1.
- When nose-down ρ2 =ρ3 <ρ1.

*Yaw Control Mechanism:* The product of reaction torque and tilt angle “∝” of Rotor 3 is used to control yaw movement. The value of the tilt angle is too small and helps to maneuver the UAV quickly. The yaw control condition is: ρ1 =ρ2 =ρ1, with ∝=0.

### 3.2. Control Algorithm

In this section the overall control hierarchy is defined to control the attitude and altitude of the tri-rotor UAV. In which we assume the desired attitude variables are $K_{1}=\;\emptyset$ (roll), $K_{2}=\;\theta$ (pitch), and $K_{3}=\;\Psi$ (yaw), respectively, and $K_{T}$ is a generalized term for the rotational angles of the system. Now the control algorithm for the attitude controlling of an actual system of the UAV is written as:

(11) $$G_{{\text{KT}}_{1}}\left(j\right)=\;\frac{\text{B }\left(j\right)}{A\left(j\right)}=Y\left(t\right)$$

The degree of the system model numerator B(j) and denominator A(j) is found to be “1” and “3”. Where $B=(B^{+}{*\text{B}}^{-})$, such that $B^{+}\text{ is }variable\;and\;B^{-}\;is\;the\;constant.$

Equation (12) presents the desired attitude response of our UAV model:

(12) $$G_{{\text{KT}}_{2}}\left(j\right)=\;\frac{B_{m}\left(j\right)}{A_{m}\left(j\right)}=Y_{m}\left(t\right)$$

Now the gradient theory which was defined by the model reference adaptive control method is implemented:

(13) $${\text{degA}}_{c}=2*\text{deg}A\left(j\right)-1=5$$

So, the RST controller will be second-ordered, and now ${\text{degA}}_{0}=\text{deg}A\left(j\right)-{\text{degB}}^{+}-1=1$.

**Remark 1.**  *For the perfect system model*$\;A_{m}\left(j\right)=\;A\left(j\right)\;and\;B_{m}\left(j\right)=B\left(j\right)$*. Where* $A_{C}\;$*and* $A_{Cm}$ *is the characteristic polynomials of the actual and desired system models and* $A_{Cm}=A_{C}$ *for the constraints and will not affect the system to change the close loop poles of the model. Otherwise,* $A_{Cm}\;$*differes the system model mismatch*.

**Fuzzy Logic Controller.** The vibrant performance of the fuzzy logic controller is described by the set of linguistic procedures that was established by a knowledgeable acquaintance in [30,31,32], in which the system “error” and variation in error rate are the input constraints, and RST are the variable outputs in our proposed controller. Formerly, RST can be improved online, using the set of rules, existing error, and variation in the error. In general, the error in the angle, combined with the mechanism output, increases. Furthermore, the controller performs well whether the error rises or the rate of error difference falls. It is important that, in the minor error phase, the earmarked control output is required to influence the change in error as soon as the error falls suddenly.

With the help of Equation (13), the degree of the proposed controller is found to be 5. The fuzzy logic-based adaptive RST controller is written in Equations (14)–(16):

(14) $$\text{FR}=q^{2}+(r_{0}\times q)+r_{1\;}$$

(15) $$\text{FS}=(s_{0}\times q^{2})+(s_{1}\times q)+s_{2}$$

(16) $$\text{FT}=\left(t_{0}\times q\times A_{0}\right)+(t_{1}\times A_{0})$$

Put the values of FR, FS, FT in the above:

(17) $$U_{{\text{FR}}_{\text{KT}}}\left(j\right)=\text{ FR}\left(e,\frac{\text{de}}{\text{dt}}\right)G_{R}\times e\left(j\right)$$

(18) $$U_{{\text{FS}}_{\text{KT}}}\left(j\right)=\text{ FS}\left(e,\frac{\text{de}}{\text{dt}}\right)G_{S}\times \text{Σe}\left(\text{ij}\right)\Delta \left(t\right)$$

(19) $$U_{{\text{FT}}_{\text{KT}}}\left(j\right)=\text{ FT}\left(e,\frac{\text{de}}{\text{dt}}\right)G_{T}\times \frac{\Delta e\left(j\right)}{\Delta t}$$

where $G_{R},\;G_{S}\;and\;G_{T}$ are the delayed control gains of the signal scaling factors.

**Remark 2.**  $G_{{\text{KT}}_{1}}\left(j\right)\text{ and}\;G_{{\text{KT}}_{2}}\left(j\right)$*, are the system models. Moreover, the system diverges at a low phase angle with an unstable control signal. The proposed control algorithm is not better in this case. Previously, in [33], a better controller was proposed for this type of case, a model having a low phase angle lying in the complex plane with some damping issues. As a result, in [34], zero cancellation in the system is situated esoteric the area which will cancel*.

**Remark 3.**  The proposed controller design at sampling of time [NT], which will remove the difficulties in the planning stage of controller implementation.

**Theorem 1.**  *After designing the control system, the next step is stability analysis, which shows the robustness level of the designed controller. The stability is highly vulnerable due to modeling errors called sensitivity. Therefore, model mismatch and model sensitivity are added in the system divergence between the actual and desired response depending upon the performance of the control system and its stability which will be taken from [34] Theorem 5.4, and examine the stability of the control system by using the model disturbance*.

**Lemma.**  *The proposed equation illustrates the close loop system model having (NT) sampling period:*

$$\frac{{\left[Y^{T}\left(t\right)\right]}^{\text{NT}}}{{\text{FR}}^{\text{NT}}}=\;\frac{{\left[G_{\text{KT}1}^{T}\left(j\right)G_{\text{KT}2}^{T}\left(j\right)H^{T,\text{NT}}\right]}^{\text{NT}}G_{\text{KT}1}^{\text{NT}}\left(j\right)\left[\frac{{\text{FT}}^{\text{NT}}}{{\text{FR}}^{\text{NT}}}\right]}{1+\;{\left[G_{\text{KT}1}^{T}\left(j\right)G_{\text{KT}2}^{T}\left(j\right)H^{T,\text{NT}}\right]}^{\text{NT}}G_{\text{KT}1}^{\text{NT}}\left(j\right)\left[\frac{{\text{FS}}^{\text{NT}}}{{\text{FR}}^{\text{NT}}}\right]}$$

$H^{T,NT\;}$is the conversion rate and will depend on the orientation of the desired signal.

**Remark 4.**  The disturbance in the pole placement method is obsolete in [34]. Since the reference model, observer polynomial, and reference model disturbance act as constraints, they are proved in convergence analysis.

**Proof of Theorem 1.**  The output of the unvarying model is considered in [35]:

$$Y^{T}\left(t\right)=G_{\text{KT}1}^{T}\left(j\right)G_{\text{KT}2}^{T}\left(j\right)H^{T,\text{NT}}\;{\left[G_{\text{KT}1}^{\text{NT}}\left(j\right)\right]}^{\text{NT}}{\left[\frac{{\text{FT}}^{\text{NT}}}{{\text{FR}}^{\text{NT}}}{\text{ FR}}^{\text{NT}}-\frac{{\text{FS}}^{\text{NT}}}{{\text{FR}}^{\text{NT}}}\;[Y^{T}\left(t\right)\right]}^{\text{NT}}{]}^{T}$$

**Figure 3:**
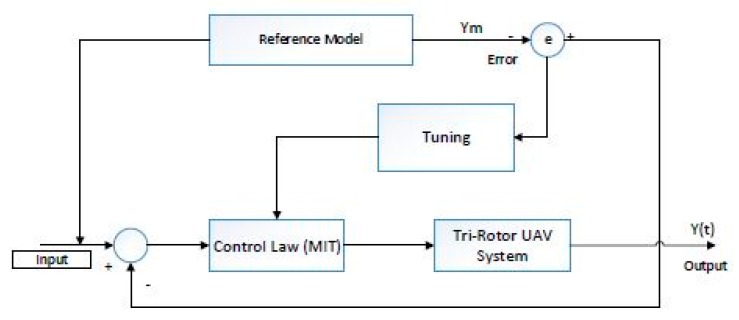
The model reference adaptive control system.

**Remark 5.**  Figure 3, gives complete work flow of proposed system using model reference adaptive control algorithm.

*Convergence*: Taking the input constraints of our controller signals gives the close loop error of the system model and unstable part of the disturbance. In other words, our proposed algorithm is able to stabilize the unstable part of the noise or disturbance model. The convergence at the desired value of parameters is done by an optimal control method based on the MIT rule to identify the errors.

**Remark 6.**  *For the proposed controller the adaptive gain is in the range of 0.15 to 5 and above this range the controller performance deteriorates.*

(20) $$e\left(j\right)=Y_{\text{actual}}\left(t\right)-Y_{m\left(\text{desired}\right)}$$

The sensitivity derivative is presented in Equation (21):

(21) $$Y\left(t\right)=\frac{B\left(j\right)T}{\left(A\left(j\right)R+B\left(j\right)S\right)\;}$$

(22) $$U_{c}\left(t\right)=\frac{A\left(j\right)R+B\left(j\right)S}{B\left(j\right)T}*\text{Y}\left(t\right)$$

The convergence proof contracts distinctly with the constraints, firstly identifying the sensitivity derivative of all of the parameters ($t_{0},{\text{ t}}_{1},r_{0},{\text{ r}}_{1},s_{0},{\text{ s}}_{1},{\text{ s}}_{2}$) of the controller.

The Diophantine equation represents as $A_{C}=A\left(j\right)R+B\left(j\right)\text{S}$ and $A_{C}=A_{0}A_{m}\left(j\right)$; therefore:

(23) $$A\left(j\right)R+B\left(j\right)S={\text{ A}}_{0}A_{m}\left(j\right)$$

The MIT rule-based sensitivity derivative of Equation (20) is:

(24) $$e\left(j\right)=\frac{B\left(j\right)T}{A\left(j\right)R+B\left(j\right)S}{*\text{U}}_{c}\left(t\right)-Y_{m}$$

**Theorem 2.**  *The model in Equation (11) with controller Equation (14–16) on the basis of system (*A(j)*,*B(j)*), having UAV model disturbance, the error of close loop output Equation (24) goes to zero asymptotically if and only if* $A_{Cm}$ = $A_{C}$*.*

**Proof of Theorem 2.**  The close loop output error *Equation* (24) is settled by$\;A_{C\;}$and compared with *Equation* (23).

1. If, and only if, $A\left(j\right)=A_{m}\left(j\right)$ and $\text{B }\left(j\right)={\text{ B}}_{m}\left(j\right)$; therefore, the close loop output error responds to e(j) and goes to zero asymptotically because $A_{\text{C}}$ is stable in Equation (13) and $A_{\text{Cm}}$ is supposed to be stable.

2. Contradiction: if $A\left(j\right)\neq A_{m}\left(j\right)$ and $\text{B }\left(j\right)\neq {\text{ B}}_{m}\left(j\right)$, the value is not cancelled by the close loop output error in Equation (24). The instability is in the denominator of e(j) if $A_{Cm}$ is unstable. Hereafter, if $A_{\text{C }}\neq A_{\text{Cm}}$ or $A_{\text{Cm}}$ is unstable, then e(j) close loop output error raises, unbounded, and it is necessary for $A_{\text{Cm}}$ to be stable to make the close loop output error zero.

Now put in the value of $\text{T}=(t_{0}q+t_{1}$)$A_{0}$ in Equation (24):

(25) $$e\left(j\right)=\frac{B\left(j\right)\left(t_{0}q+t_{1}\right)A_{0}}{A\left(j\right)R+B\left(j\right)S}{*\text{U}}_{c}\left(t\right)-(Y_{m})$$

Therefore, w.r.t $“t_{0}”$, the partial derivative of Equation (25) is:

(26) $$\{\begin{array}{c} \frac{\delta \text{e}\left(j\right)}{\delta (t_{0})}=\frac{B\left(j\right){\text{qA}}_{0}}{\left(A\left(j\right)R+B\left(j\right)S\right)}*U_{c}\left(t\right)\\ \frac{\delta \text{e}\left(j\right)}{\delta \left(t_{0}\right)}=\left(\frac{B\left(j\right){\text{qA}}_{0}}{\left(A\left(j\right)R+B\left(j\right)S\right)}\right)*\frac{A\left(j\right)R+B\left(j\right)S}{B\left(j\right)T}*Y\left(t\right)\\ \frac{\delta \text{e}\left(j\right)}{\delta \left(t_{0}\right)}=\;\frac{A_{0}q}{T}*Y\left(t\right) \end{array}$$

The partial derivative of Equation (25) w.r.t ${“\text{t}}_{1}”$ is:

(27) $$\{\begin{array}{c} \frac{\delta \text{e}\left(j\right)}{\delta (t_{1})}=\left(\frac{B\left(j\right)}{\left(A\left(j\right)R+B\left(j\right)S\right)}\right)*U_{c}\left(t\right)\;\\ \frac{\delta \text{e}\left(j\right)}{\delta (t_{1})}=\left(\frac{B\left(j\right)}{\left(A\left(j\right)R+B\left(j\right)S\right)}\right)*\frac{A\left(j\right)R+B\left(j\right)S}{B\left(j\right)T}*Y\left(t\right)\\ \frac{\delta \text{e}\left(j\right)}{\;\delta (t_{1})}=\frac{A_{0}}{T}*Y\left(t\right) \end{array}$$

From Equation (24), replace the value of R: (R = ${\left(q\right)}^{2}+r_{0}q+r_{1}$)

(28) $$e\left(j\right)=\frac{B\left(j\right)T}{A\left(j\right)\left(q^{2}+r_{0}q+r_{1}\right)+B\left(j\right)S}{*\text{U}}_{c}\left(t\right)-(Y_{m})$$

Partial differentiate Equation (25) w.r.t $“r_{0}”$:

(29) $$\{\begin{array}{c} \frac{\delta \text{e}\left(j\right)}{\delta \left(r_{0}\right)}=-\left(\frac{B\left(j\right)\text{TAS}}{{\left(A\left(j\right)R+B\left(j\right)S\right)}^{2}}\right)*U_{c}\left(t\right)\\ \frac{\delta \text{e}\left(j\right)}{\delta \left(r_{0}\right)}=-\left(\frac{B\left(j\right)\text{TAS}}{{\left(A\left(j\right)R+B\left(j\right)S\right)}^{2}}\right)*\frac{A\left(j\right)R+B\left(j\right)S}{B\left(j\right)T}*Y\left(t\right)\\ \frac{\delta \text{e}\left(j\right)}{\delta \left(r_{0}\right)}=-\frac{AS}{A_{0}A_{m}\left(j\right)}*Y\left(t\right) \end{array}$$

Partial differentiate Equation (25) w.r.t ${“\text{r}}_{1}”$:

(30) $$\frac{\delta \text{e}\left(j\right)}{\delta (r_{1})}=-\frac{A\left(q\right)\;}{A_{0}A_{m}\left(j\right)}*\text{Y}\left(t\right)$$

Put the value of S, $S=(s_{0}{*\text{q}}^{2}+s_{1}*\text{q}$ + $s_{2})$ in Equation (24):

(31) $$e\left(j\right)=\frac{B\left(j\right)T}{A\left(j\right)R+B\left(j\right)\left(s_{0}q^{2}+s_{1}q+s_{2}\right)}{*\text{U}}_{c}\left(t\right)-(Y_{m})$$

Now the Partial derivative of Equation (31) w.r.t $“s_{0}”:$

(32) $$\{\begin{array}{c} \frac{\delta e\left(j\right)}{\delta (s_{0})}=-\frac{\left(B{\left(j\right)}^{2}{*\text{q}}^{2}\right)}{{\left(A\left(j\right)R+B\left(j\right)S\right)}^{2}}{*\text{U}}_{c}\left(t\right)\\ \frac{\delta e\left(j\right)}{\delta (s_{0})}=-\frac{\left(B{\left(j\right)}^{2}{*\text{q}}^{2}\right)}{{\left(A\left(j\right)R+B\left(j\right)S\right)}^{2}}\frac{A\left(j\right)R+B\left(j\right)S}{B\left(j\right)T}*\text{Y}\left(t\right)\\ \frac{\delta e\left(j\right)}{\delta (s_{0})}=\;-\frac{B\left(j\right){*\text{q}}^{2}}{A_{0}A_{m}\left(j\right)}*\text{Y}\left(t\right) \end{array}$$

Now taking Partial derivative of Equation (31) w.r.t $“s_{1}”$

(33) $$\frac{\delta \text{e}\left(j\right)}{\delta \left(s_{1}\right)}=-\frac{B\left(j\right)\text{q }}{\left(A_{m}\left(j\right)A_{0}\right)}*\text{Y}\left(t\right)$$

Likewise, Partial derivative of the Equation (31) w.r.t $“s_{2}”$ gives

(34) $$\frac{\delta \text{e}\left(j\right)}{\delta \left(s_{2}\right)}=-\frac{B\left(j\right)}{\left(A_{m}\left(j\right)A_{0}\right)}*\text{Y}\left(t\right)$$

By applying the MIT algorithm in the desired model of the system, where Equations (35) and (36) denote the cost function which is based on the MIT rule. Whitaker demonstrates the difference in system bounds as a function of the system error and the gradient of the system error with respect to the system constraints and takes the partial derivative of the gradient error with respect to its constraints. The constraints of the particular model with the initial estimate J(KT), and the rate of change of speed among the desired and actual model is taken from [36]. To minimize the error with respect to time so that the desired response is achieved requires an optimum control with cost function.

**Theorem 3.**  *In [20] the least square estimation* $J\left(\bar{KT}\right)\;$*and their polynomial characteristic solution depends upon the Diophantine equation and stability of*$\;A_{Cm}$.

**Proof of Theorem 3.**  The solution also depends upon the Diophantine equation as well as the stability of$\;A_{Cm}$. By using Theorem 2:

1. If e(j)→0 as l→∞ and leading $J\left(\bar{KT}\right)=0$, the solution gives the smallest positive value of cost function.
2. $A_{Cm}$ is a contradiction, if it is not stable from Theorem 2; e(j)⇸0 as l→∞, thus $J\left(\bar{KT}\right)\neq 0$. This is not optimal because, as seen in step 1 of the proof, there exists a solution that makes$\;J\left(\bar{KT}\right)=0$.
  (35) $$J\left(\text{KT}\right)=\frac{1}{2}e^{2}\left(\text{KT}\right)$$
  (36) $$\frac{\text{dKT}}{\left(\text{dt}\right)}=\;-\gamma^{\prime }\frac{l}{l_{o}}{\text{ y}}_{\left(m\right)}e=\;-{\gamma *\text{y}}_{\left(m\right)}*\text{e}\left(j\right)$$

After that, apply the MIT rule to derivate control variables ($s_{0},{\text{ s}}_{1},{\text{ s}}_{2},{\text{ r}}_{0},{\text{ r}}_{1},{\text{ t}}_{0},{\text{ and t}}_{1})$ and set in the controller gives

(37) $$\frac{d\left(s_{0}\right)}{\text{dt}}=\;-\gamma_{e}\frac{\delta \text{e}}{{\delta \text{s}}_{0}}\;\frac{d\left(s_{0}\right)}{\text{dt}}=\;\frac{\gamma_{e}q^{2}B\left(j\right)Y\left(t\right)}{A_{m}\left(j\right)A_{0}}$$

(38) $$\frac{d\left(s_{1}\right)}{\text{dt}}=\;-\gamma_{e}\frac{\delta \text{e}}{{\delta \text{s}}_{1}}\;\frac{d\left(s_{1}\right)}{\text{dt}}=\;\frac{\gamma_{e}B\left(j\right)\text{qY}\left(t\right)\;}{A_{m}\left(j\right)A_{0}}$$

(39) $$\frac{d\left(s_{2}\right)}{\text{dt}}=\;-\gamma_{e}\frac{\delta \text{e}}{{\delta \text{s}}_{2}}\;\frac{d(s_{2})}{\text{dt}}=\;\frac{\gamma_{e}B\left(j\right)Y\left(t\right)\;}{A_{m}\left(j\right)A_{0}}$$

(40) $$\frac{d(r_{0})}{\text{dt}}=\;-\gamma_{e}\frac{\delta \text{e}}{{\delta \text{r}}_{0}}\;\frac{d(r_{0})}{\text{dt}}=\frac{\gamma_{e}A\left(j\right)\text{qY}\left(t\right)\;}{A_{m}\left(j\right)A_{0}}$$

(41) $$\frac{d(r_{1})}{\text{dt}}=\;-\gamma_{e}\frac{\delta \text{e}}{{\delta \text{r}}_{1}}\;\frac{d(r_{1})}{\text{dt}}=\frac{\gamma_{e}A\left(j\right)Y\left(t\right)\;}{A_{m}\left(j\right)A_{0}}$$

(42) $$\frac{d\left(t_{0}\right)}{\text{dt}}=\;-\gamma_{e}\frac{\delta \text{e}}{{\delta \text{t}}_{0}}\;\frac{d(t_{0})}{\text{dt}}=-\frac{\gamma_{e}B\left(j\right)\text{q }}{A_{0}}$$

(43) $$\frac{d\left(t_{1}\right)}{\text{dt}}=\;-\gamma_{e}\frac{\delta \text{e}}{{\delta \text{t}}_{1}}\;\frac{d(t_{1})}{\text{dt}}=-\frac{\gamma_{e}}{\left(A_{m}\left(j\right)\right)}$$

Now, the main controller equation becomes:

$$U_{F-{\text{Hybrid}}_{\emptyset ,\;\theta ,\Psi }}=\left(\frac{\left(F\left(T_{0}q+T_{1}\right)A_{0}\right)}{F(q^{2}+r_{0}q+r_{1})}\right)*(\text{Uc}\left(t)\right)-\left(\frac{F(S_{0}q^{2}+S_{1}q+S_{2})}{F\left(q^{2}+r_{0}q+r_{1}\right)}\right)Y\left(t\right)$$

The change in UAV orientation depends upon the rate of change of the control commands, which makes the system respond quite better towards stability by using our proposed algorithm.

The linguistics levels of the fuzzy hybrid controller are assigned as (BN) below negative, (SN) small negative, (ZR) zero, (SP) small positive, and (BP) big positive. The fuzzy logic controller if-then rules are defined in Table 2, Table 3 and Table 4, such that error “e” is the rotor speed having range in −10 to +10, the derivative error range is −5 to +5, and the output range of the fuzzy hybrid controller is 0 to 1 with R = 0.667, S = 0.5, and T = 0.5, respectively.

**Figure 4:**
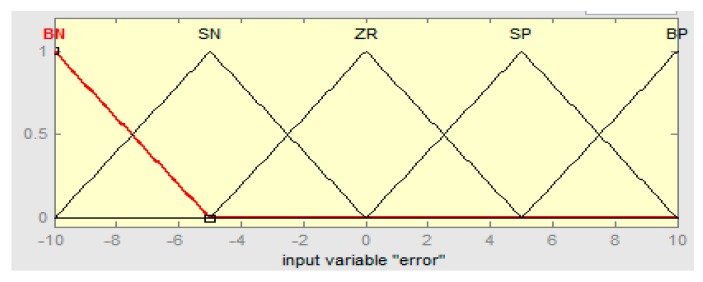
Input error membership function of fuzzy Logic.

**Figure 5:**
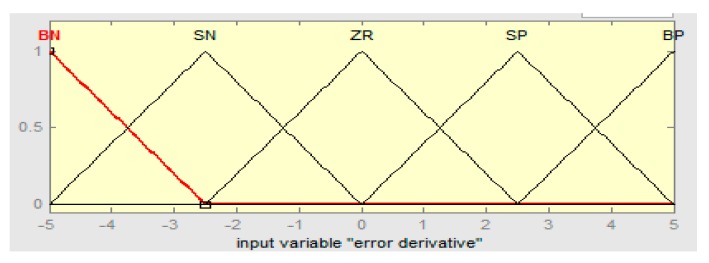
Input derivative error membership function of fuzzy logic.

**Figure 6:**
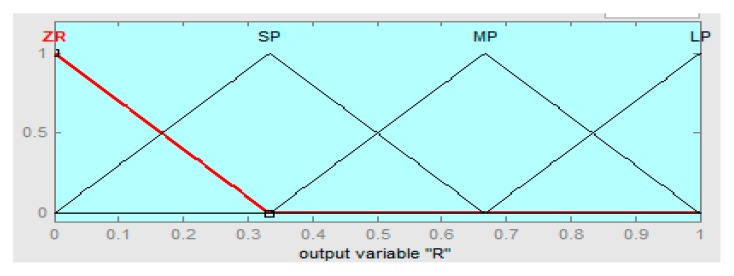
Regulation output gain membership function of fuzzy logic.

**Figure 7:**
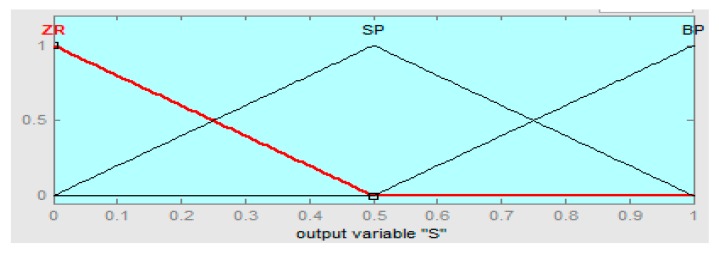
Pole-placement output gain membership function of fuzzy logic.

**Figure 8:**
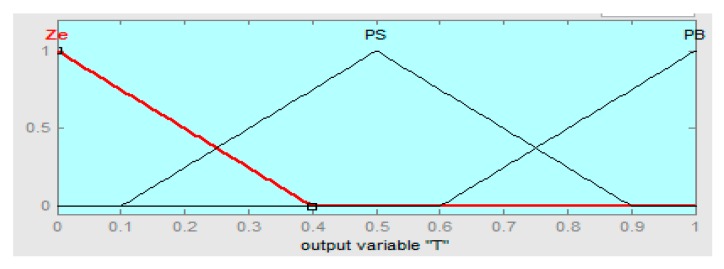
Tuning the output gain membership function of fuzzy logic.

The input error membership function of the fuzzy logic controller (FLC) is shown in Figure 4. Figure 5 shows the input derivative error of FLC. Moreover, Figure 6, Figure 7 and Figure 8 show the output gains of RST-based FLC logic.

## 4. Simulation Results and Discussions

The validity and robustness of our proposed algorithm are presented in this section. Moreover, the nonlinear simulations for the stabilization of tri-rotor parameters are shown in Table 5. All of the simulations were done via Simulink, MATLAB. In all simulations, we compared our proposed fuzzy-hybrid controller with the adaptive RST controller of [20].

Ideally, we can say that there are two subsystems of the UAV; one is rotational, while the other is a translational velocity response. The rotational subsystem is responsible for controlling the initial errors, thereby stabilizing the attitude of UAV and converges to zero. Pitch, roll, and yaw angles are also converging to zero to realize the hovering state. The rotational subsystem and Euler angles do not depend on translational components. However, the translational components depend on Euler angles.

**Figure 9:**
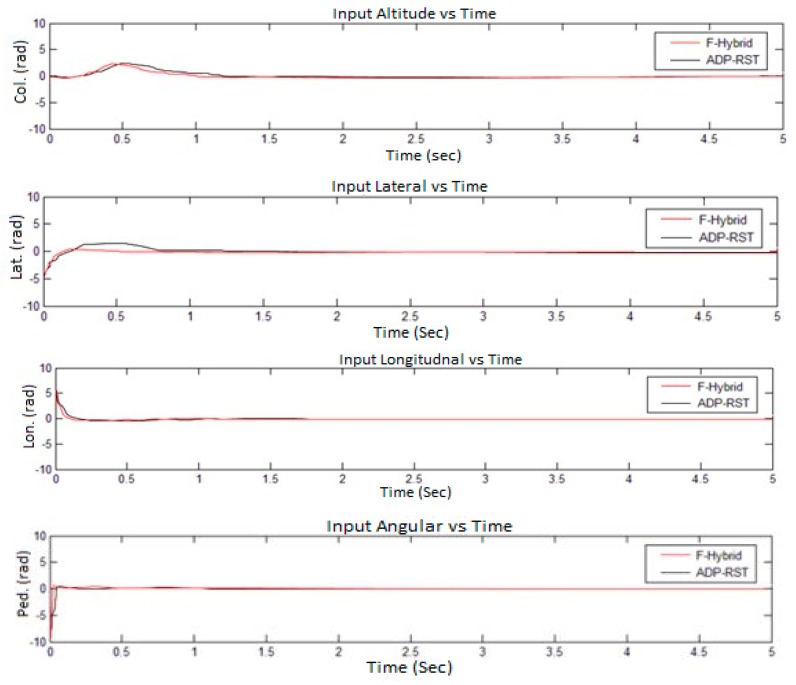
Comparison of F-Hybrid with adaptive RST for control commands.

**Figure 10:**
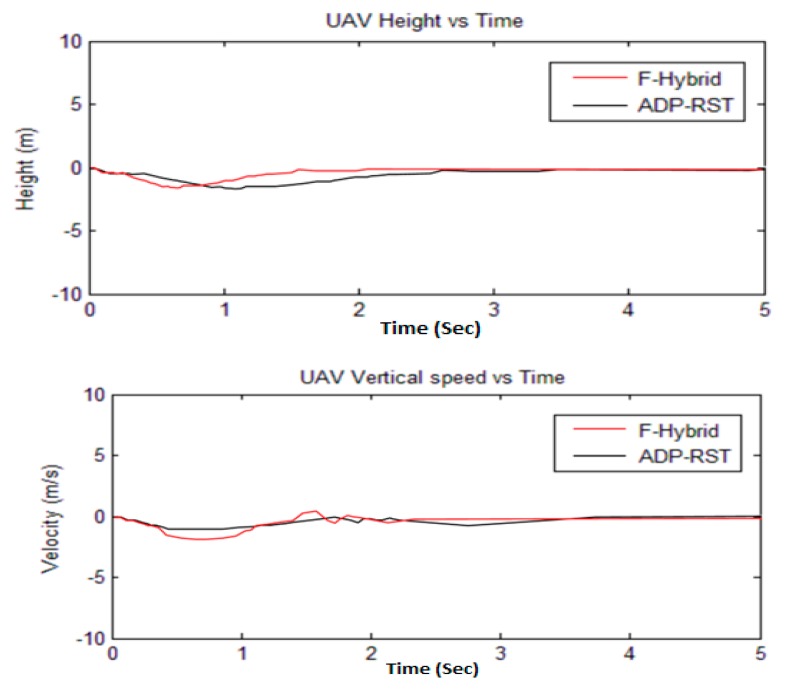
Comparison of F-Hybrid with adaptive RST for height and vertical speed.

The four control commands, *i.e.*, altitude, lateral, longitudinal, and angular, are shown in Figure 9. The settling and rise time are within one to one and a half seconds for each input channel along with zero overshoot. By comparing the adaptive RST controller control commands, it is not perfectly linear because in the adaptive RST controller the control commands do not perfectly converge to zero and have undershoot and overshoot, initially, which causes the UAV to dislocate from the desired position due to the presence of errors. However, our proposed method completely converges to zero and exactly reaches the perfect location as shown in Figure 10, which shows the actual reached height and vertical speed response of the UAV.

**Figure 11:**
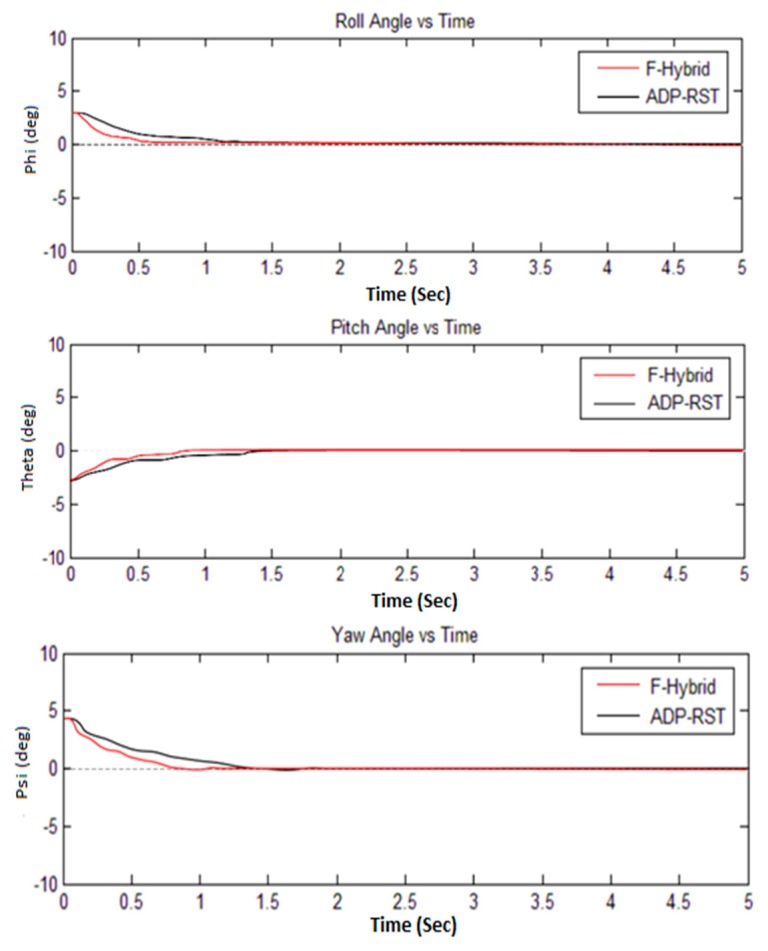
Comparison of F-Hybrid with adaptive RST for Euler angle response.

**Figure 12:**
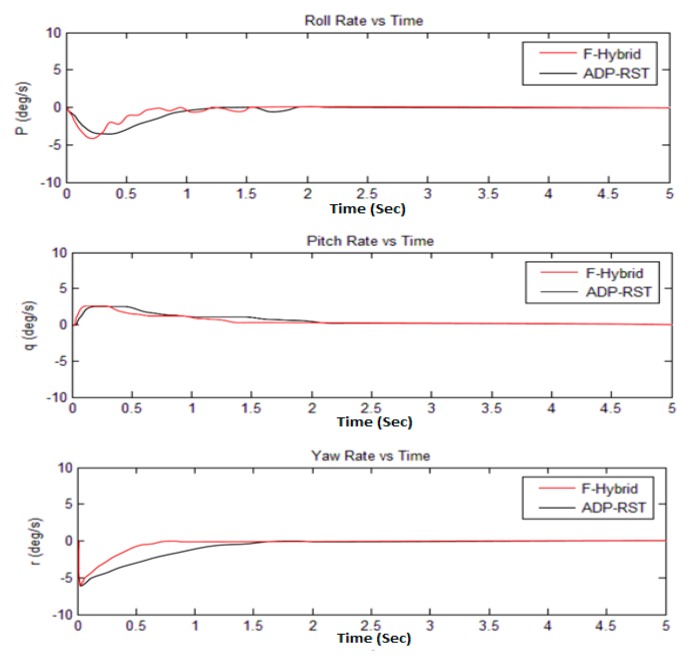
Comparison of F-Hybrid with adaptive RST for rotational velocity responses.

**Figure 13:**
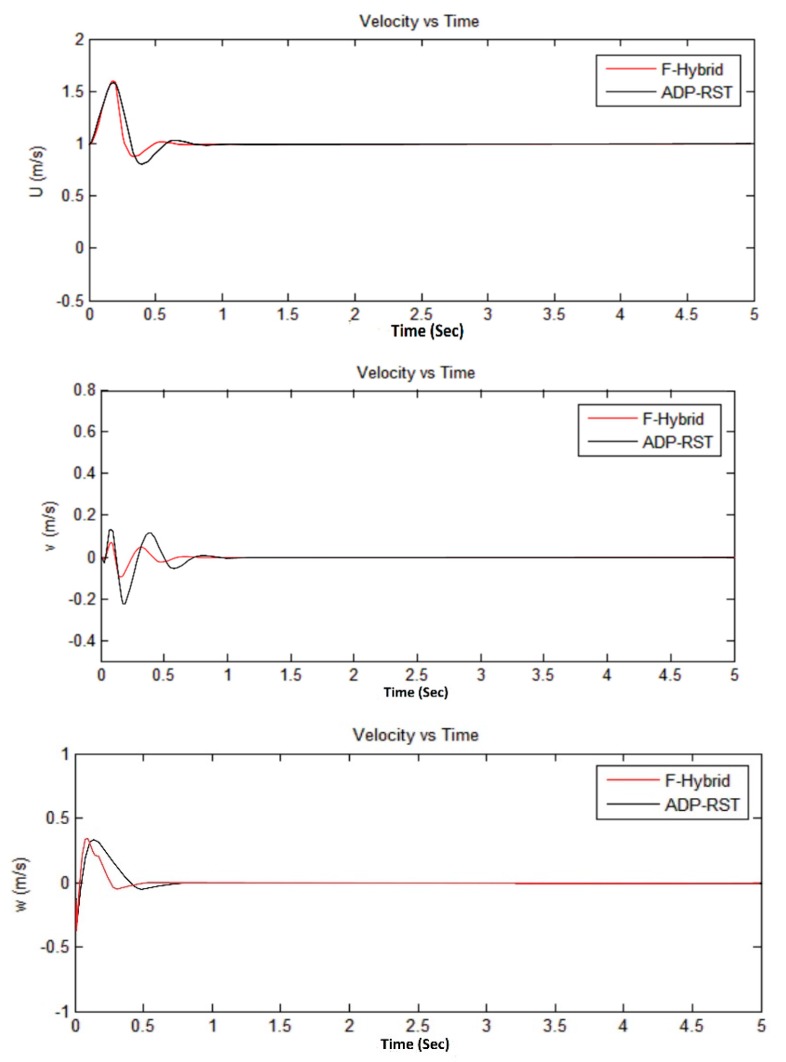
Comparison of F-Hybrid with adaptive RST for translational velocity responses.

The Euler angular responses are shown in Figure 11; one can observe that initial angles are non-zero, but it will converge to zero, which means that it will perform the attitude hold control by comparing with the adaptive RST controller. Figure 12 shows the rotational rate responses and, by comparing the transitional rate responses given in Figure 13, observe that there are several “bumps” but ultimately converge to the desired value. In Figure 12 and Figure 13, our proposed algorithm converges to zero at about one second and have no settling time error, but the adaptive RST controller settled at about two to two and a half seconds.

The sampling time was set to 0.2 s for all simulations, whereas every simulation was done in five seconds. The simulation results from Figure 9, Figure 10, Figure 11 and Figure 12 show that our proposed algorithm is quite fast and converges to zero without any settling time error. The Euler angle initial conditions are ∅=2. 5, θ=−2, and ψ = 4.7 degrees. In Figure 13, the translational velocity components are U = 1, V = W = 0, meaning it can easily control the attitude of UAV, without any overshoot, undershoot, and transient errors. Furthermore, the settling time response also gets better than the previously-used adaptive RST controller. In a very short time of about 1 to 1.5 s, all of the simulated parameter values yield to the original trimmed conditions. The results show that our proposed controller is able to stabilize the attitude angles and altitude of the tri-rotor UAV. 

## 5. Conclusions

In this paper, a new and novel approach of a fuzzy hybrid controller is presented for stabilizing the nonlinear behavior of a tri-rotor UAV and to achieve the desired altitude. The model of the tri-rotor UAV is based on the Newton-Euler method. The method is applied on the translational and rotational velocity subsystems of the aircraft. To observe the stability of the UAV we must stabilize the attitude and altitude responses. The RST with MRAC nonlinear controller algorithm is based on the MIT rule and the sensitivity function of the closed-loop system has shown significant results. Moreover, fuzzy logic controllers have been proposed with linguistic logic, which enhanced the performance. The effectiveness and stability of our algorithm are implemented using nonlinear simulation and it is observed that the proposed method has better transient characteristics and performance with zero steady state error on rigid environments with reasonable rise and settling time. Furthermore, the proposed method shows better robustness and very fast convergence towards stability in the presence of a system disturbance.

## Figures and Tables

**Table 1:** Dynamic constants of a tri-rotor UAV.

x, y, z Axis System	Roll (φ)	Pitch (θ)	Yaw (ψ)
Aerodynamic Force Components	X	Y	Z
Aerodynamic Moment Components	L	M	N
Translational Velocity	U	V	W
Angular Rates	p	q	r
Three-Axis Inertia	I_x_	I_y_	I_z_

**Table 2:** Fuzzy logic controller If-Then rule for “R”.

$\vec{de/dt}$	BN	SN	ZR	SP	BP
error↓					
BN	ZR	SP	MP	MP	SP
SN	SP	SP	SP	MP	MP
ZR	SP	MP	MP	MP	MP
SP	SP	SP	MP	SP	SP
BP	ZR	SP	MP	LP	S

**Table 3:** Fuzzy logic controller If-Then rule for “S”.

$\vec{de/dt}$	BN	SN	ZR	SP	BP
error↓					
BN	ZR	ZR	SP	BP	BP
SN	ZR	SP	SP	SP	BP
ZR	ZR	SP	SP	SP	BP
SP	ZR	SP	SP	SP	BP
BP	ZR	ZR	SP	SP	BP

**Table 4:** Fuzzy Logic Controller If-Then rule for “T”.

$\vec{de/dt}$	BN	SN	ZR	SP	BP
error↓					
BN	SP	SP	ZR	ZR	ZR
SN	BP	SP	SP	SP	BP
ZR	BP	SP	SP	SP	BP
SP	BP	SP	SP	SP	SP
BP	BP	BP	BP	SP	SP

**Table 5:** Tri-rotor parameters.

Parameters	Values	Si Units
Ix	0.3105	kg·m^2^
Iy	0.2112	kg·m^2^
Iz	0.2215	kg·m^2^
l	0.3050	m
Mass	0.785	kg
